# Effect of Liothyronine Treatment on Dermal Temperature and Activation of Brown Adipose Tissue in Female Hypothyroid Patients: A Randomized Crossover Study

**DOI:** 10.3389/fendo.2021.785175

**Published:** 2021-11-19

**Authors:** Betty Ann Bjerkreim, Sara Salehi Hammerstad, Hanne Løvdal Gulseth, Tore Julsrud Berg, Sindre Lee-Ødegård, Anbjørg Rangberg, Christine Monceyron Jonassen, Helen Budge, David Morris, James Law, Michael Symonds, Erik Fink Eriksen

**Affiliations:** ^1^ Department of Endocrinology, Morbid Obesity and Preventive Medicine, Oslo University Hospital, Oslo, Norway; ^2^ Institute of Clinical Medicine, Faculty of Medicine, University of Oslo, Oslo, Norway; ^3^ Endocrinology, Pilestredet Park Specialist Center, Oslo, Norway; ^4^ Department of Pediatrics, Oslo University Hospital, Oslo, Norway; ^5^ Department of Chronic Diseases and Ageing, Norwegian Institute of Public Health, Oslo, Norway; ^6^ Department of Transplantation, Oslo University Hospital, Oslo, Norway; ^7^ Center for Laboratory Medicine, Østfold Hospital Trust, Grålum, Norway; ^8^ Department of Chemistry, Biotechnology and Food Science, Norwegian University of Life Sciences, Ås, Norway; ^9^ Academic Child Health, School of Medicine, University of Nottingham, Nottingham, United Kingdom; ^10^ Bioengineering Research Group, Faculty of Engineering, University of Nottingham, Nottingham, United Kingdom; ^11^ Nottingham Digestive Disease Centre and Biomedical Research Centre, School of Medicine, University of Nottingham, Nottingham, United Kingdom; ^12^ The Faculty of Dentistry, University of Oslo, Oslo, Norway

**Keywords:** hypothyroidism, brown adipose tissue (BAT), cold-induced thermogenesis, infrared thermography, liothyronine, levothyroxine

## Abstract

**Background:**

Thyroid hormones are essential for the full thermogenic response of brown adipose tissue (BAT) and have been implicated in dermal temperature regulation. Nevertheless, persistent cold-intolerance exists among a substantial proportion of hypothyroid patients on adequate levothyroxine (LT4) substitution.

**Materials and Methods:**

To assess if skin temperature and activation of BAT during treatment with liothyronine (LT3) differs from that of LT4 treatment, fifty-nine female hypothyroid patients with residual symptoms on LT4 or LT4/LT3 combination therapy were randomly assigned in a non-blinded crossover study to receive monotherapy with LT4 or LT3 for 12 weeks each. Change in supraclavicular (SCV) skin temperature overlying BAT, and sternal skin temperature not overlying BAT, during rest and cold stimulation were assessed by infrared thermography (IRT). In addition, abundance of exosomal miR-92a, a biomarker of BAT activation, was estimated as a secondary outcome.

**Results:**

Cold stimulated skin temperatures decreased less with LT3 *vs*. LT4 in both SCV (mean 0.009°C/min [95% CI: 0.004, 0.014]; *P*<0.001) and sternal areas (mean 0.014°C/min [95% CI: 0.008, 0.020]; *P*<0.001). No difference in serum exosomal miR-92a abundance was observed between the two treatment groups

**Conclusion:**

LT3 may reduce dermal heat loss. Thermography data suggested increased BAT activation in hypothyroid patients with cold-intolerance. However, this finding was not corroborated by assessment of the microRNA biomarker of BAT activation.

**Clinical Trial Registration:**

ClinicalTrials.gov, identifier NCT03627611

## Introduction

Brown adipose tissue (BAT) plays an important role in adaptive thermogenesis through non-shivering mechanisms (1). Energy in BAT is dissipated as heat due to activation of the unique uncoupling mechanism mediated by the mitochondrial uncoupling protein 1 (UCP1) (2–4). Thyroid hormones activate BAT both centrally *via* the sympathetic nerve system (5), and peripherally with direct stimulation of UCP1 expression in the mitochondria in hypothermic rodents (6, 7).

It is estimated that 5-10% of hypothyroid patients continue to complain of residual symptoms including persistent cold-intolerance (8), suggesting intracellular thyroid hormone deficiency despite adequate levothyroxine substitution based on serum thyroid stimulating hormone (TSH). Further, it is still controversial whether these patients experience alleviation of symptoms when switching to a combination therapy with levothyroxine (LT4) and liothyronine (LT3) (9). The demonstration of a specific polymorphism in deiodinase 2 has indeed contributed to the debate that LT4 may not be a sufficient therapy in all patients with hypothyroidism, as a result of potential reduced conversion of T4 to intracellular T3 in target cells (10).

Since 45% of the intracellular T3 in BAT is derived from the circulation (11), reduced levels of free T3 in patients with hypothyroidism may result in a lower body temperature due to impaired BAT activation. Accordingly, it may be hypothesized that persisting cold-intolerance in hypothyroid patients on LT4 substitution, may be related to reduced T3 effects on BAT. Some studies have suggested improvement in quality of life in hypothyroid patients on LT4/LT3 combination therapy (12–15), while other studies failed to demonstrate such effects (16–21).

BAT is found in the neck, along the spine and around internal organs (22–25). Currently, BAT can only be reliably visualized using prolonged cold exposure of fasted subjects followed by 18F-FDG PET/CT (22, 26). This technique is, however, less well suited for large-scale studies with repeated measurements due to both radiation exposure and cost. In recent years thermal imaging has emerged as a valid alternative (27, 28). In addition, a study by Chen et al. demonstrated that the concentrations of a specific micro-RNA, miR-92a, elicited from BAT exosomes in humans, were inversely correlated to BAT activation measured by 18F-FDG PET/CT (29).

In addition to activation of BAT, thyroid hormones also affect body temperature by stimulating obligatory thermogenesis (30, 31). Moreover, in a study of T3 receptor defective mice, Warner et al. linked BAT activation and effects of T3 on the vasculature (32, 33). They found T3 receptor defective animals to have a lower body temperature, pointing to a defect in heat conservation. The lower temperature was caused by insufficient peripheral vasoconstriction due to impaired adrenergic action on blood vessels causing increased heat loss over the tail surface of the animals. The authors considered the heat loss to be the trigger of additional thermogenesis by BAT (33). Based on these findings, we wanted to test the hypothesis that patients with residual hypothyroid symptoms, despite biochemical euthyroidism on LT4 therapy, exhibit abnormal dermal heat loss and BAT activation as a sign of deficient T3-receptor action.

We therefore compared skin temperature and BAT activation on LT3 and LT4 therapy by evaluating differences in supraclavicular (BAT) and sternal skin (non-BAT) temperatures measured with infrared thermography (IRT). Levels of serum exosome miR-92a were further measured as a marker for activation of BAT in the two groups.

## Materials and Methods

### Ethics

The study was approved by the Regional committee for ethics in medical research (ref. no. 2017/1883) and by the Norwegian Medicines Agency (ref. no. 18/02175). The study was registered at ClinicalTrials.gov (NCT03627611) and performed in accordance with the Declaration of Helsinki. Written informed consent was obtained from all patients prior to participation in the study. The study was carried out between June 2018 and June 2020 at the Department of Endocrinology, Morbid Obesity and Preventive Medicine, Oslo University Hospital.

### Patients

Female patients with hypothyroidism, who were referred to the endocrine outpatient clinic at Oslo University Hospital and endocrinologists in private practice in Oslo were recruited. In addition, information about the study was posted in the Norwegian Thyroid Association`s membership magazine. Interested patients were encouraged to contact the study team for further information and a screening visit. Inclusion criteria were: women with primary hypothyroidism and residual symptoms such as fatigue, impaired cognition or persistent cold-intolerance despite replacement therapy with LT4 or combination LT4/LT3 therapy for at least 6 months; aged 18-65 years; and serum TSH 0.1-3.6 mU/L. Exclusion criteria were: pregnancy or breast-feeding; cardiovascular disease; other endocrinological disease; chronic liver or kidney disease; other hormonal substitution therapy except for levothyroxine, liothyronine and desiccated thyroid extract; use of antidepressants, antipsychotic or anxiolytic medications or use of beta blockers.

The presence of residual symptoms was assessed by a questionnaire consisting of ten stated hypothyroid symptoms, including cold-intolerance, fatigue, cognitive disturbances, emotional disturbances, edemas, dry skin, menstrual disorders, weight gain, hair loss and constipation, where patients answered “yes” or “no” to each statement. If a patient answered “yes” to three or more statements, they were considered to meet inclusion criteria.

**Figure 1:**
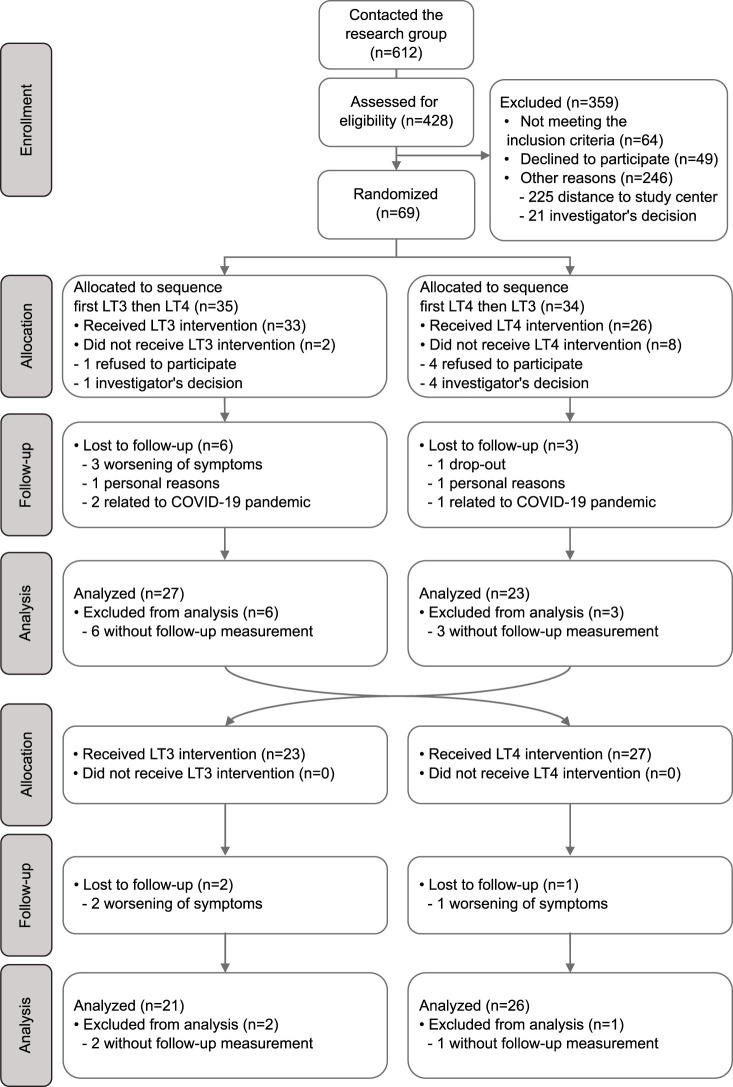
Flow chart describing recruitment, randomization and analysis of patients in the study.

Sixty-nine female patients were recruited and randomized. However, ten patients dropped out before treatment initiation, either due to a change in the patient’s, wish to participate (five patients) or due to the investigator’s, decisions (three patients expressed an unwillingness to take study medicine according to protocol and two patients communicated that they would withdraw if they did not experience effect of treatment). They were therefore excluded from all analyses (**Figure 1**). Thus, a total of 59 patients were randomized.

### Study-Design

The study was a randomized crossover study with patients randomly assigned to receive either LT4 or LT3 for 12 weeks, followed by a crossover for another period of 12 weeks. 1:1 block randomization with randomly selected block sizes of 4, 6 and 8 was performed. Patients were not blinded to treatment. If a patient was already on LT3/LT4 combination therapy, LT3 monotherapy or desiccated thyroid extract before study start, they had to complete a 4-week run-in period on LT4 monotherapy. We estimated the LT4 dose by calculating 15 μg of LT3 as equal to 50 μg of LT4, based on a pharmacoequivalence study which showed that the LT4/LT3 equivalence ratio is approximately 3:1 (34). LT3 treatment was started at a dose of one third of the patient’s previous LT4 dose. LT4 treatment were maintained at the patient’s usual dose. A wash-out period was not included between the two treatment periods due to the shorter half-life of LT3 compared to LT4 and a relatively long treatment period of 12 weeks. After attending the initial study visit, patients were advised to take LT4 treatment once daily, half an hour before breakfast, and LT3 treatment thrice daily, half an hour before, or two hours after, a meal.

Thyroid function tests were performed every four weeks during the study period and treatment doses were adjusted, if necessary, aiming to achieve serum TSH of 0.1-1.5 mU/L.

Patients were supplied with study drugs in boxes containing 100 tablets. To ensure compliance, patients were asked to return all remaining tablets and empty boxes at each study visit for counting of tablets.

### Study Protocol

Patients underwent evaluation at baseline and after both treatment periods. All study visits were conducted at the Diabetes and Metabolism Research Laboratory, Oslo University Hospital. On each study visit, patients reported to the laboratory between 0800 and 1000 h after an overnight fast of at least 8 hours including abstinence from nicotine, caffeine and other stimulants. The patients were told not to consume alcohol or perform strenuous exercise the last 48 hours before study visits and avoid applying water, lotion or perfume at the neck and upper chest area the morning of test visits. On arrival, patients were immediately required to lie in a study bed.

### Anthropometrics

Height and weight were measured with patients wearing light clothing and no shoes using a stadiometer (Seca, Hamburg, Germany) and a class III digital weighing scale (Tanita BWB-800A, Tanita Corporation, Tokyo, Japan), respectively. Body mass index (BMI) was calculated as bodyweight in kilograms divided by the square of the height in meters.

### Hormone analyses

Serum TSH (reference range: 0.5 - 3.6 mU/l) was analyzed at The Hormone Laboratory, Oslo University Hospital using a non-competitive immunofluorometric analysis by Autodelfia (Wallac Oy, Turku, Finland).

### Indirect Calorimetry

Energy expenditure (EE) was assessed by indirect calorimetry and performed using a Jaeger Oxycon Pro (Erich Jaeger, Viasys Healthcare, Germany) computerized flow-through canopy gas analyzer system. Gas and volume were calibrated before each measurement, and ambient conditions were registered at each visit. EE was measured while patients were resting in a supine position under a blanket, to prevent shivering, with a canopy hood placed over the patient´s head. After a 10-minute adaption period, the expired and inspired air was continuously measured for 20 minutes in steady-state. Whole body substrate oxidation was estimated based on the mean values of VO_2_ and VCO_2_ measurements. No shivering was observed and no patient reported shivering during the procedure.

### Infrared Thermography (IRT)

We performed IRT to assess changes in skin temperature in the supraclavicular (SCV) fossa overlying BAT and a sternal region not overlying BAT as previously reported by Law et al. (35). Immediately following the indirect calorimetry measurements patients were seated in a relaxed and upright position looking straight ahead. The upper chest area and the neck region were exposed while patients underwent thermographic analysis using a FLIR® A310 thermal imaging camera (FLIR Systems, Wilsonville, OR, USA) with thermal resolution 320 x 240 pixels, <0.05°C sensitivity and ± 2°C accuracy. Emissivity was set to 0.98. The camera was positioned at the level of the neck and at a 1 m distance, fastened onto a tripod. Images were acquired every minute for five minutes (resting period) before patients were exposed to cold by placing both hands into two 5-L receptacles containing cold water (mean temperature 15.1 ± 0.53°C). A further 5 minutes of images (stimulation period) were acquired at the same rate of one per minute. This protocol was in accordance with other studies evaluating the use of IRT in assessment of BAT in terms of short acclimatization period (36, 37), rapid cold stimulation consisting of hands immersed in water (38–40), acquisition of images each minute during the cold stimulation (39, 41) and highlight the region of interest (ROI) using a specific scale (39, 42, 43). No patient reported pain during the procedure. Mean room-temperature was 21.7 ± 1.7°C.

**Figure 2:**
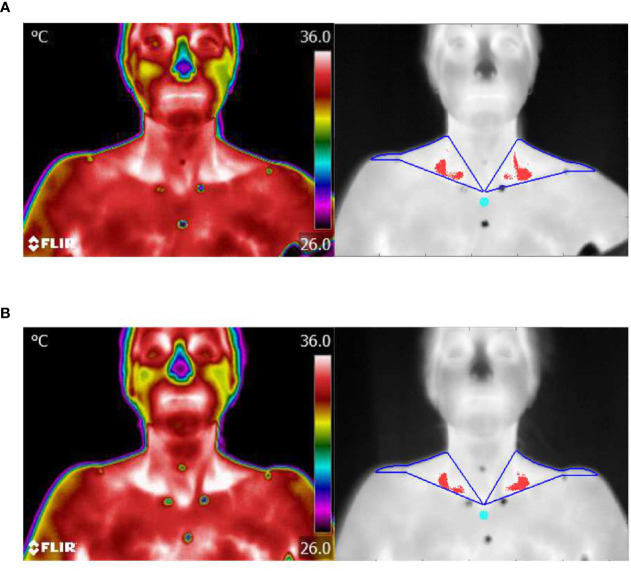
Thermal imaging. **(A)** Representative example of thermal images of the skin area overlying neck and upper chest during cold stimulation after 12 weeks of LT4 treatment with corresponding images of the contour of ROI (blue) and hottest 10% of pixels (red) identified in the SCV fossa and sternal reference region (turquoise). **(B)** Same as for **(A)**, but after 12 weeks of LT3 treatment.

Images were saved in FLIR’s proprietary JPEG format. The radiometric data were converted to temperature data and the images analyzed within MATLAB (2017b, MathWorks, Natick, MA, USA) as described by Law et al. (27). Skin temperatures overlying the SCV fossa bilaterally and a sternal region (as reference) in the upper chest just superior to the sternal angle were determined for each image by manually plotting five points representing the apices of the SCV ROI and one point representing apex of the 10-pixel circular sternal reference region on the sternum (**Figures 2A, B**). Temperature points within the ROIs were analyzed to identify the hottest 10%, corresponding to BAT (27) and the SCV temperature was defined as the median temperature of the hotspot. Additionally, the median temperature of the circular sternal reference region was calculated.

### Core Temperature

At the baseline visit, all patients were given a Braun High Speed Thermometer PRT 1000 and asked to record their morning rectal temperature once a week throughout the 24-week study period in a standardised diary. Mean temperature was calculated for each treatment period of 12 weeks.

### Levels of Serum Exosome miR-92a

Fasting blood samples from all patients were collected in serum tubes without a separating gel, centrifuged for ten minutes at room temperature within 30 minutes, aliquoted and stored at -80°C. Intact exosomes were isolated from 200 µl serum using Total Exosome Isolation reagent (cat no. 4478360, ThermoFisher Scientific, Waltham, MA, USA) according to the manufacturer’s instructions. The isolated exosomes were kept at -20°C until RNA isolation. Total RNA was isolated and purified using the Total Exosome RNA and Protein isolation Kit (cat no. 4478545, ThermoFisher Scientific) according to the manufacturers’ instructions. Isolated RNA was stored at -80°C prior to analysis. TaqMan MicroRNA Assays (cat no. 4427975, ThermoFisher Scientific, Waltham, MA, USA) was used to quantify hsa-miR-92a (miRBase ID: 000430) according to the TaqMan Small RNA Assay user guide. U6snRNA (miRBase ID: 715680) was used for normalization, as described by Chen et al. (29).

Briefly, 5 µl total RNA was reverse transcribed using TaqMan™ MicroRNA Revers Transcription Kit (Cat no. 4366596, ThermoFisher Scientific, Waltham, MA, USA) in two separate reactions, using specific RT primers supplied with the TaqMan MicroRNA Assay. Reverse transcription was performed as follows: 16°C for 30 minutes, 42°C for 30 minutes and 85°C for 10 minutes. The cDNA was stored for up to one week at -20°C before Real-time PCR. Real time PCR for miR-92a and U6snRNA were performed on 1.3 µl of the corresponding cDNA using TaqMan™ Fast Advanced Master mix (cat no. 4444558, ThermoFisher Scientific, Waltham, MA, USA), and specific TaqMan MicroRNA assays on QuantStudio™7 Flex Real-time PCR system with the following thermocycling protocol: 50°C for 20 minutes, 95°C for 20 seconds, 40 times of 95°C for 1 second and 60°C for 20 seconds. cDNA synthesis and PCR reactions for both assays were performed in the same set-up for each sample. For data analyses, the expression levels of miR-92a were determined using the 2^-ΔCt^ method, normalized to U6snRNA. All miR-92a samples were analysed at the Østfold Hospital Trust.

### Sample Size and Power Calculations

Based on estimations of change in skin temperature with a minimal clinical difference of 0.4°C and standard deviation of 0.7°C, we calculated that a sample size of 51 would be sufficient to detect a significant difference with 80% power and α=0.05 between the two study treatments. Allowing for a 15% loss to follow-up, a study population of 60 patients was set as the recruitment target.

### Statistical Analyses

Baseline data were analyzed by one-way ANOVA for variables with normal distribution, Mann-Whitney U test for variables without normal distribution, and Chi-square test for categorical variables. All variables were tested for normality by quantile-quantile (QQ)-plots and histogram. Statistical significance was defined as two-tailed *P <*0.05.

Mixed linear models were created to estimate the effects of cold stimuli (time in minutes) on the slope reflecting the change in supraclavicular and sternal area temperatures (in degree Celsius). Random intercept for participants and random slopes for treatment groups displayed the lowest Bayesian information criterion (BIC), as compared to simpler mixed models. For outcomes measured only twice for each participant within each treatment period, such as body weight, energy expenditure and core temperature, mixed models with only random intercepts for participants could be constructed. All mixed models included additive effects of treatment period and sequence order, so to adjust for potential period and carry-over effects. Sensitivity analyses were performed by re-constructing the mixed models including additive effects of relevant covariates such as BMI, outdoor and room temperature. Time curves were visualized as box and whiskers plots (mean, interquartile ranges, min/max and outliers’ points).

Mediation analysis of miR-92a on resting SCV minus sternal area temperature after each treatment period was performed using the R package *mediation* (Version 4.5.0) with 1000 permutations and *set.seed (1)* to ensure reproducibility. The null model was the mixed model, as described above. Next, a model was constructed substituting the outcome with the mediator of interest. Finally, a model was constructed similarly to the null model, but including the mediator of interest as a covariate. The final model may lead to the following interpretations: (1) if the effect of LT3 therapy on relative SCV temperature is reduced and becomes insignificant, the mediator may fully explain the LT3 effect on relative SCV temperature; (2) if the effect of LT3 therapy on relative SCV temperature is reduced, but remains significant, the mediator only partially explain the LT3 effect on relative SCV temperature; and (3) if the effect of LT3 therapy on relative SCV temperature remains the same despite the presence of the potential mediator, then miR-92a do not mediate the LT3 effect on relative SCV temperature.

Statistical analysis was carried out using R (Version 4.0.2; R Core Team, 2016) and the R-packages *car* (Version 3.0.8) and *Ime4* (Version 1.1.23). The R-packages *ggplot2* (Version 3.3.2) and *patchwork* (Version 1.1.0) were used for visualizations.

## Results

There were no differences in baseline characteristics between patients allocated to receiving LT4 first *vs*. LT3 first (**Table 1**). Fifty-four patients (91.5%) reported cold-intolerance as a persistent hypothyroid symptom, despite TSH values within the normal range (**Table 1**). Further, study conditions were similar at test visits after 12 weeks treatment with LT4 and LT3 in terms of room- and water temperature. Of the 59 patients randomized and allocated to treatment, 47 patients (79.7%) completed both treatment periods (**Figure 1**).

**Table 1:** Baseline characteristics.

	Prior to randomization (n = 59)	Allocated to first LT4 then LT3 (n = 27)	Allocated to first LT3 then LT4 (n = 32)	*P* value*
Age (years)	42.9	42.8 ± 8.6	42.9 ± 10.7	0.99
Age at hypothyroidism diagnosis (years)	30.6 ± 10.2	29.8 ± 8.6	31.2 ± 11.4	0.59
Duration of substitution monotherapy LT4 (years)	10.6 ± 7.0	10.9 ± 7.3	10.3 ± 6.8	0.78
Type of therapy at inclusion				
LT4 monotherapy	46 (78.0)	20 (74.1)	26 (81.3)	0.51
LT4/LT3 combination therapy	12 (20.3)	7 (25.9)	5 (15.5)	0.73
Thyroid extract	1 (1.7)	0 (0)	1 (3.1)	1.00
Etiology of hypothyroidism				
Autoimmune/ idiopathic	56 (94.9)	27 (100%)	29 (90.6)	0.30
Post-surgical	2 (3.4)	0 (0)	2 (6.3)	0.55
Radioiodine	1 (1.7)	0 (0)	1 (3.1)	1.00
Body mass index (kg/m^2^)	28.1 ± 5.6	28.5 ± 5.9	27.8 ± 5.5	0.62
TSH (mU/L)	0.64 (0.26-1.60)	0.82 (0.28-1.50)	0.63 (0.25-1.9)	0.77
FT4 (pmol/L)	16.8 (14.7-19.0)	17.0 (14.2-20.0)	16.4 (14.8-18.0)	0.97
FT3 (pmol/L)	4.4 (3.8-4.9)	4.4 (3.8-4.8)	4.4 (3.8-5.0)	0.66
Positive TPO-ab^1^	31 (52.5)	17 (63.0)	14 (43.8)	0.23
Any residual hypothyroid symptoms				
Fatigue	57 (96.6)	26 (96.3)	31 (96.6)	1.00
Cold-intolerance	54 (91,5)	26 (96.3)	28 (87.5)	0.46
Cognitive disturbances	48 (81.4)	24 (88.9)	24 (75.0)	0.30
Emotional disturbances	38 (64.4)	16 (59.3)	22 (68.8)	0.63

Data are presented as mean ± SD or number (%) or median (interquartile range: 25-75%) as appropriate.

*P values for analysis of differences between the two treatment groups.

TSH, thyroid stimulating hormone (normal range 0.5-3.6); FT4, free thyroxine (8.0-21.0); FT3, free triiodothyronine (2.8-7.0); TPO-ab, thyroid peroxidase antibodies. ^1^Cut-off value for positive TPO-ab was 35 kIU/l.

Median weekly (IQR 25-75%) doses of study drug administered were 775 µg (658-950) for LT4 and 245 µg (210-280) for LT3. Both the LT4 and LT3 doses had to be adjusted a mean 0.7 times during the 12-week treatment period.

### Serum TSH Levels

We observed a higher TSH level after LT3 compared to LT4 treatment (median 1.33 mU/L (interquartile range (IQR) 0.47-2.26) *vs*. median 0.61 mU/L (IQR 0.25-1.20); *P*=0.018).

### Clinical Parameters and Energy Expenditure

**Figure 3:**
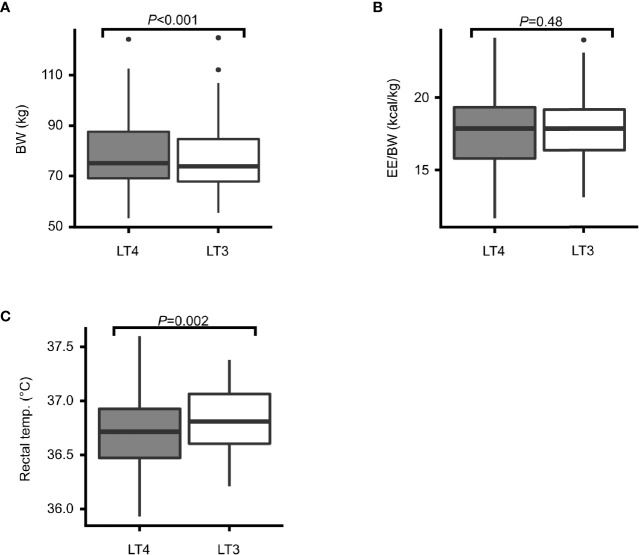
Body weight, energy expenditure and core temperature. Box plots showing treatment effects after 12 weeks treatment with LT4 (grey) and LT3 (white) in terms of: **(A)** body weight (BW) in kilograms, **(B)** energy expenditure relative to body weight (EE/BW), and **(C)** rectal temperature in degrees Celsius.

Body weight was significantly lower after 12 weeks of treatment with LT3 compared to LT4 (mean -1.07 kg [95% CI: -0.50, 1.63]; *P*<0.001) (**Figure 3A**).

Energy expenditure (EE) relative to body weight did not differ between the two treatment groups (mean 0.11 kcal/day [95% CI: -0.41, 0.197]; *P*=0.48) (**Figure 3B**).

There was a small, but highly statistically significant difference in mean core temperature between the two treatment groups with higher temperature during 12 weeks of LT3 treatment compared to LT4 treatment (mean 0.08°C [95% CI: 0.03, 0.13]; *P*=0.002) (**Figure 3C**).

### Infrared Thermal Imaging (IRT)

**Figure 4:**
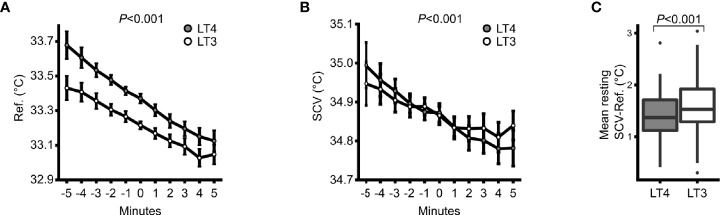
Infrared thermography. **(A)** Response in sternal reference region temperature to cold stimuli on LT4 (grey) and LT3 (white) treatment. Minutes -5 to 0 depicts the response to room air and minutes 0 to 5 the response to hand immersion of both hands into cold water. **(B)** Same as for **(A)**, but for supraclavicular (SCV) temperature. Data are means ± 95% confidence intervals. **(C)** A box & whiskers plot for resting SCV temperature minus sternal reference temperature within each of the two treatment periods.

In response to cold stimuli, the cooling effect was lower on LT3 *vs*. LT4 for both the SCV (mean 0.009 [95% CI: 0.004, 0.014] °C per minute; *P*<0.001) and the sternal areas (mean 0.014 [95% CI: 0.008, 0.020] °C per minute; *P*<0.001) (**Figures 4A, B**), with a steeper drop in cold stimulated temperatures on LT4. Mean resting SCV temperature relative to sternal temperature was 0.14 [95% CI: 0.03, 0.26] °C higher on LT3 *vs*. LT4 therapy (*P*<0.001) (**Figure 4C**). Thermal images after cold stimulation showed a smaller and less intense white-coloured area in SCV regions on LT4 (**Figure 2A**) compared to LT3 (**Figure 2B**) which reflects lower temperature in the first situation.

### Micro-RNA

We found no differences in serum exosomal miR-92a abundance (2^-ΔCt^) between the two treatment groups (ß 20.13 [95% CI: -6.07, 46.91]; *P*=0.13) (**Table 2**) and concentrations had no mediating effect on resting SCV minus sternal temperature (<0.1% mediated effect; *P*=0.960).

**Table 2:** Effects of LT3 *vs*. LT4 mediation analysis of miR-92a on resting supraclavicular (SCV) temperature minus sternal reference temperature.

	Beta coefficient	95% confidence interval	*P* value
Resting SCV temp – ref temp	0.15	0.024, 0.284	0.02
miR-92a	20.13	-6.07, 46.91	0.13
Resting SCV temp – ref temp, corrected for miR-92a	0.153	0.022, 0.285	0.02

## Discussion

This is the first study to specifically compare the effect of liothyronine (LT3) *vs*. levothyroxine (LT4) on skin temperature and activation of brown adipose tissue (BAT) assessed by infrared thermography and microRNA in hypothyroid patients with residual symptoms. Skin temperatures in both sternal and supraclavicular skin areas decreased more during cold exposure on LT4 therapy compared to LT3, despite higher TSH levels. We also observed a significant increase in body temperature during LT3 treatment compared to LT4 treatment, which supports our thermal imaging results. However, the difference was only 0.1°C thus the clinical significance is debatable.

Our findings are in agreement with our hypothesis that LT3 reduces dermal heat loss considerably more than LT4. The elevated skin temperature in the resting period on LT4 therapy is consistent with the results reported by Warner et al. where T3 receptor defective mice showed increased skin temperature because of heat loss over the tail surface due to vasodilatation resulting in lower body temperature (32, 33). Our results may therefore reflect deficient activation of T3 receptors in hypothyroid patients with persistent cold-intolerance on LT4 therapy resulting in inappropriate heat dissipation in sternal skin area in a thermoneutral state. The combination of decreased activation of BAT and increased heat loss from skin on LT4 therapy compared to LT3 could further exacerbate the feeling of cold in these patients. Unfortunately, our two different evaluation methods of BAT by thermography and microRNA are not consistent. Thus, the results pertaining to BAT activation remain equivocal. To our knowledge, the current study is, nevertheless, the only one implementing analysis of this specific micro-RNA from serum exosome as an indicator of BAT activation, using U6snRNA as reference gene, as proposed by Chen et al. (29). Therefore, the analysis should be considered as an addition to the literature on miR-92a and the results must be interpreted with caution. Anyhow, our results being similar to results obtained in mice with defective T3 receptors (33), suggest that patients with residual symptoms on LT4 monotherapy or LT4/LT3 combination therapy in fact display deficient T3 action at the receptor level in vascular cells.

The elevated body temperature in combination with a significant weight loss on LT3 therapy suggest increased metabolic rate in the LT3 group. Other reasons for increased adaptive thermogenesis, such as diet or muscle shivering, may be alternative explanations to cold-induced effects, causing the observed effects on body temperature and weight. Interestingly, there was no simultaneous increase in energy expenditure demonstrable in calorimetry, although TSH levels were higher than in the LT4 group. This paradox may be explained by the fact that weight loss in humans is usually accompanied by a decrease in total energy expenditure (44). Other reasons may be that although all patients were instructed in how to relate to diet, physical activity and alcohol prior to the examinations, we cannot exclude that being in a particular treatment group may have affected the behavior leading to bias and skewed results. Alternatively, we lacked power to identify a true change of small magnitude.

Thyroid hormones stimulate obligatory thermogenesis in many tissues in addition to the cold-induced adaptive thermogenesis in BAT (45), and it is possible that minor differences in obligatory thermogenesis may explain the differences in core temperature and body weight. Despite this, LT3 treatment also resulted in less cooling in both the SCV skin area and sternal reference skin area during cold stimulation, implying that the subjects on LT3 retained heat better than subjects on LT4. This is consistent with our hypothesis that LT3 activates T3-receptors leading to vasoconstriction to a greater extent than LT4 in patients with residual hypothyroid symptoms.

This study has some limitations. We conducted the study using an open-label design which may have affected the outcome due to both nocebo and placebo effects resulting in skewed and biased data. We aimed to minimize this potential effect by objectively measuring hard end points, but acknowledge some effects may still remain, e.g., with respect to weight changes.

Possible disadvantages of the crossover design of the current study include the following: 1) the order in which treatments are administered, may lead to a sequence effect that can affect the outcome; 2) a carry-over effect between treatments may also confound the treatment effects, but may be mitigated by a sufficiently long washout period. However, we tested for both sequence and carry-over effects in our linear mixed model and found no such effects.

Since we observed a significant difference in TSH levels in addition to skin temperatures between the two treatment groups, we cannot exclude a potentially eliminating effect of the former. However, TSH levels after both LT3 and LT4 therapy were within the normal range in addition to study target level. We therefore consider this difference to be clinically insignificant.

Activity of BAT is inversely correlated with body fat (22, 23, 25, 46). Since mean BMI among our study patients was in the overweight range (47), thickness of the subcutaneous tissue overlying the SCV region may have reduced emitted infrared radiation due to insulation. However, since we compared skin temperatures in the SCV fossa to a sternal reference area, the insulating effect of obesity was probably minor.

Infrared thermography is only able to measure skin temperature. SCV temperature after a cold exposure may therefore partly be explained by other thermogenic tissues including skeletal muscles and blood vessels, in addition to BAT.

In conclusion, we have demonstrated that liothyronine reduces the drop in skin temperature during cold stimulation in both supraclavicular and sternal areas, and increases body temperature. Severe cold-intolerance is common in hypothyroid patients on levothyroxine therapy with residual symptoms, as in our study participants. This is consistent with the notion that intracellular T3 receptors are not sufficiently activated in hypothyroid patients with persistent cold intolerance on LT4 therapy. Our data suggest that patients with residual hypothyroid symptoms despite optimal LT4 dosing actually exhibit objective differences when treated with LT3 versus LT4. We therefore propose that LT3 may improve cold-intolerance in patients with residual hypothyroid symptoms on LT4 therapy. However, long-term studies are needed to assess the long-term safety before LT3 monotherapy can be considered an alternative treatment regimen in these patients.

## Data Availability Statement

The raw data supporting the conclusions of this article will be made available by the authors, without undue reservation.

## Ethics Statement

The studies involving human participants were reviewed and approved by the regional committee for medical and health research ethics in South-Eastern Norway. The patients/participants provided their written informed consent to participate in this study.

## Author Contributions

Conceived and designed the experiments: BB, SH, HG, and EE. Performed the experiments: BB, SL-Ø, and EE. Contributed materials/analysis tools: TB, AR, CJ, HB, DM, JL, and MS. Wrote the paper: BB, SH, HG, TB, SL-Ø, AR, CJ, HB, DM, JL, MS, and EE. All authors contributed to the article and approved the submitted version.

## Funding

This study has been supported by grants from the Grant number: (2017101) and the Grant number: (1/2020).

## Conflict of Interest

The authors declare that the research was conducted in the absence of any commercial or financial relationships that could be construed as a potential conflict of interest.

## Publisher’s Note

All claims expressed in this article are solely those of the authors and do not necessarily represent those of their affiliated organizations, or those of the publisher, the editors and the reviewers. Any product that may be evaluated in this article, or claim that may be made by its manufacturer, is not guaranteed or endorsed by the publisher.
